# Submicroscopic chromosomal imbalances contribute to early abortion

**DOI:** 10.1186/s13039-018-0386-0

**Published:** 2018-07-21

**Authors:** Haibo Li, Minjuan Liu, Min Xie, Qin Zhang, Jingjing Xiang, Chengying Duan, Yang Ding, Yinghua Liu, Jun Mao, Ting Wang, Hong Li

**Affiliations:** Center for Reproduction and Genetics, The Affiliated Suzhou Hospital of Nanjing Medical University, NO. 26 Daoqian Street, Suzhou, 215002 Jiangsu Province China

**Keywords:** Chromosomal abnormality, Abortion, Chorionic villus, Karyotype analysis

## Abstract

**Background:**

Chromosomal abnormalities are one of the genetic mechanisms associated with abortion. However, the roles of submicroscopic chromosomal imbalances in early abortion are still unclear. This study aims to find out whether submicroscopic chromosomal imbalances contribute to early abortion.

**Methods:**

A total of 78 chorionic villus specimens from early spontaneous abortion patients with no obvious abnormality are collected after miccroassay analysis (the case group). At the same time, 60 chorionic villus specimens from induced abortion patients with no obvious abnormality are selected as the control group. The submicroscopic structures of chromosomes from two groups are analyzed using an array-based comparative genomic hybridization (aCGH).

**Results:**

In the case group, 15 specimens show submicroscopic chromosomal abnormalities including 14 micro-deletion/micro-duplication in chromosomes 2, 4, 5, 6, 7, 8, 9, 12, 15, 16, 18, and 22, and 1 uniparental disomy (UPD) in chromosome 19. Moreover, no pathogenic copy number variations are found in the control group. The results between these two groups exhibit significantly statistical difference.

**Conclusion:**

Submicroscopic chromosomal imbalances may be one of the main reasons for early abortion.

**Electronic supplementary material:**

The online version of this article (10.1186/s13039-018-0386-0) contains supplementary material, which is available to authorized users.

## Background

Spontaneous abortion, also known as miscarriage, means the loss of a pregnancy before 20 weeks and the weight of fetal less than 1000 g, which is the natural death of an embryo before it can survive independently [1]. Risk factors for miscarriage include an older parent, previous miscarriage, drug or alcohol use, diabetes, exposure to tobacco smoke, and obesity, and so on [2]. Early abortion that occurs in the first 12 weeks of pregnancy, constitutes about 80% of miscarriages [3]. A large proportion of early abortion cases is caused by chromosomal abnormalities. Among them, half of embryonic miscarriages have an aneuploidy, namely aberrant number of chromosomes [4].

About 6–13% stillbirth is related to karyotypic alterations [5–7]. Karyotype analysis is reported to detect numerical abnormalities of chromosomes like triploid, haploid and polyploidy and structural abnormality like translocation and inversion. Therefore, the technique of G-binding karyotype analysis is usually used to diagnose the karyotype abnormality of tissues in the dead fetus [8]. However, due to weak cell viability of died fetus and difficult culture of cells, only 45–65% of cells can be obtained for the diagnosis of cytogenetics [9]. Recent researches have found that submicroscopic structure copy number variations (CNVs) is the genetic etiology of fetal growth retardation, miscarriage, stillbirth and other congenital diseases, which can be detected by the technique of chromosomal microarray analysis (CMA) [10, 11].

CMA, also called molecular karyotyping analysis, includes array-based comparative genomic hybridization (aCGH), and single nucleotide polymorphism (SNP) array [12, 13]. By far, CMA has been used in the research and diagnosis of tumors [14], neurologic and mental diseases [15–17], and congenital diseases [18, 19], etc. However, there are few reports about CMA analysis in miscarriage and stillbirth. In the present study, in order to determine whether submicroscopic chromosomal imbalances are the main reasons for early abortion CMA is used to analyze and compare the submicroscopic structures of chromosomes in chorionic villus specimens from both the early spontaneous abortion group and the induced abortion group.

## Methods

### Patients

A total of 78 chorionic villus specimens from early spontaneous abortion patients with no obvious abnormality (the case group) and 60 chorionic villus specimens from induced abortion patients with no obvious abnormality (the control group) were collected by the Center for Reproduction and Genetics (The affiliated Suzhou Hospital of Nanjing Medical University, Suzhou, Jiangsu, China) after karyotype analysis. All experiments were carried out with the approval of the Institutional Ethics Commission.

### Array-CGH

The 8*60 k aCGH analysis (Agilent Technologies, Santa Clara, CA, custom-designed) was performed according to the manufacturer’s protocols (version 4.0, Agilent Technologies, CA, USA). Briefly, 0.5 μg of genome DNA (gDNA) was digested with an enzyme mixed with 2.5 U Alu I and 2.5 U Rsa I (SureTag DNA Labeling Kit, Agilent Technologies, CA, USA) at 37 °C for 2 h. gDNA was labeled with Cy3 and Cy5 (test and control, respectively), and further purified with SureTag DNA Labeling Kit (Agilent Technologies, CA, USA) following the instructions provided by the company. The 60 K whole genome aCGH chip containing one oligoprobe/8 kb (Agilent Technologies) was hybridized with labeled DNA at 65 °C for 24 h.

### Image and data analysis

The hybridized chip was scanned, and images were quantified with FEATURE EXTRACTION software (Agilent Technologies). Data were normalized using a vendor-provided equation (log2 [Cy3/Cy5] 0.25). CNVs selection was conducted in Agilent CytoGenomics (Agilent Technologies), followed by a filter to select regions with three or more adjacent probes and a minimum average log2 ratio + 0.25.

## Result

The results of aCGH show deletion as well as duplication in different genomic regions affected early spontaneous abortion. In the case group, 15 specimens with chromosomal abnormalities including 14 micro-deletion/micro-duplication and 1 uniparental disomy for chromosome 19 are found among 78 specimens (Table 1). The length of abnormality of 15 specimens ranges from 380 kb to 82.08 mb. The aberrations are unequally distributed among the 15 patients, for example, patient 14 has 4 aberrations. Furthermore, the number of CNVs in patients 14 and 15 are higher than that of other patients (Table 1). The CNV plots of one patient are shown in Additional file 1: Figure S1 as a representative. Conversely, no pathogenic CVNs are found in the control group.

**Table 1:** The results of aCGH in 15 patients

Patients	The results of aCGH	Number of CNVs
1	Chr16: (76,320,001–90,160,000) X3,13.84 M, 16q terminal duplication syndrome; Chr18: (56,020,001–78,020,000) × 1, 22 M, 18q deletion syndrome.	2 (35.84)
2	Chr 6p25.3-p22.3 (347,038–17,543,199) x1, 17.20 M deletion syndrom.	1 (17.20)
3	Chr 19 uniparental disomy.	
4	Chr22: (18,900,001–21,420,000) X1, 2.52 M, 22q11 deletion syndrome (Velocardiofacial/DiGeorge syndrome).	1 (2.52)
5	Chr22: (18,880,001–21,460,000) X1, 2.58 M, 22q11 deletion syndrome (Velocardiofacial / DiGeorge syndrome).	1 (2.58)
6	Chr22: (35,420,001–39,100,000)X1, 3.68 M, Waardenburg syndrome.	1 (3.68)
7	Chr22: (18,880,001–21,820,000) X1, 2.94 M, 22q11 deletion syndrome (Velocardiofacial / DiGeorge syndrome).	1 (2.94)
8	Chr9: (130,900,001–133,080,000) X1, 2.18 M.	1 (2.18)
9	Chr7: (64,680,001–65,200,000) X1, 520 K, deletion syndrome; Chr12: 160,001–34,820,000) X3, 34.66 M, 12p duplication syndrome.	2 (35.18)
10	Chr16: (76,320,001–90,160,000) X3, 13.84 M, 16q terminal duplication syndrome; Chr18: (56,020,001–78,020,000) X1, 22 M, 18q deletion syndrome.	2 (35.84)
11	Chr15: (22,740,001–29,120,000) X1, 6.38 M, Prader-Willi syndrome, Angelman syndrome.	1 (6.38)
12	Chr18: (66,540,001–68,040,000) X3, 1.5 M, duplication, pathogenicity unknown; Chr18: (68,760,001–77,400,000) X3, 8.64 M, 18q terminal duplication syndrome.	2 (10.14)
13	Chr16: (76,260,001–90,160,000) X3, 13.9 M, 16q terminal duplication syndrome; Chr18: (56,080,001–78,020,000) X1, 21.94 M, 18q deletion syndrome.	2 (35.84)
14	Chr2: (1–24,020,000) X3, 24.02 M, 2p partial trisomy syndrome; Chr5: (19,080,001–19,460,000) X1, 380 K, deletion, pathogenicity unknown; Chr8: (160,001–8,100,000) X1, 7, 94 M, deletion syndrome Cornelia de Lange syndrome; Chr8: (12,540,001–24,660,000) X3, 12.12 M, duplication; pathogenicity unknown.	4 (44.46)
15	Chr2: (160,940,001–243,020,000) X3, 82.08 M, 2q terminal duplication syndrome; Chr4: (184,020,001–190,940,000) X1, 6.92 M, 4q terminal deletion syndrome.	2 (89)

Those chromosomal aberrations are not equally distributed in all chromosomes. The submicroscopic chromosomal abnormalities occur in chromosome 2, 4, 5, 6, 7, 8, 9, 12, 15, 16, 18, and 22 (Table 2). Among them, the submicroscopic chromosomal abnormalities of chromosome 4, 8, 9, 18, and 22 have ever been reported by others. A total of 9 amplification events and 14 deletion events are invovled in 15 patients. Twenty-one pathogenic CNVs including Waardenburg syndrome, Velocardiofacial/DiGeorge syndrome, Prader-Willi syndrome, Angelman syndrome, and Cornelia de Lange syndrome are found. 22q11 (18900001–21,420,000) occurs 2.52 M deletion syndrome, which leads to Velocardiofacial/DiGeorge syndrome. Besides, the pathogenicity of two CNVs on chromosomes 5 and 8 are still unknown. Amplifications frequently are identified in chromosomes 2, 16, 18 (77.78%), and chromosomal deletions are more frequent in chromosomes 18 and 22 (50.00%). The total frequency of submicroscopic chromosomal abnormalities appearing in chromosomes 2, 16, 18, and 22 is 60.87% (Table 3).

**Table 2:** The results of aCGH per chromosome

Chromosome	Patients	The results of aCGH	References	Number of CNVs
2	14, 15	14: chr2, 24.02 M 2p partial trisomy syndrome pathogenicity unknown; 15: chr2, 82.08 M 2q terminal duplication syndrome.	–	2
4	15	Chr4, 6.92 M 4q terminal deletion syndrome.	[27]	1
5	14	Chr5, 380 K deletion, pathogenicity unknown.	–	1
6	2	Arr6, 17.20 M deletion syndrome.	–	1
7	9	Chr7, 520 K deletion syndrome.	–	1
8	14	Chr8, 7.94 M deletion syndrome, Cornelia de Lange syndrome; Chr8, 12.12 M duplication, pathogenicity unknown.	[28]	1
9	8	Chr9, 2.18 M, deletion syndrome.	[29]	1
12	9	Chr12, 34.66 M 12p duplication syndrome.	–	1
15	11	Chr15, 6.38 M, deletion syndrome, Prader-Willi syndrome, Angelman syndrome.	–	1
16	1, 10, 13	1: chr16, 13.84 M terminal duplication syndrome; 10: chr16, 13.84 M 16q terminal duplication syndrome; 13: chr16, 13.9 M 16q terminal duplication syndrome.	–	3
18	1, 10, 12, 13	1: chr18, 22 M deletion syndrome; 10: chr18, 22 M 18q deletion syndrome; 12: chr18, 1.5 M 18q terminal duplication, pathogenicity unknown; chr18, 8.64 M 18q terminal duplication syndrome; 13: chr18,21.94 M 18q deletion syndrome.	[30]	4
19	3	Chr 19 uniparental disomy.	–	1
22	4, 5, 6, 7	4: chr22, 2.52 M 22q11 deletion syndrome (Velocardiofacial/DiGeorge syndrome); 5: chr22, 2.58 M 22q11 deletion syndrome (Velocardiofacial/DiGeorge syndrome); 6: chr22, 3.68 M deletion syndrome Waardenburg syndrome; 7: chr22, 2.94 M 22q11 deletion syndrome (Velocardiofacial / DiGeorge syndrome).	[31]	4

“-” means no investigation by others

**Table 3:** Number and frequency of aberrations per chromosome

Chromosome	Duplication(Patients)	Frequency (%)	Deletions (Patients)	Frequency (%)	Total Frequency(%)
2	14,15	22.22	–		8.70
4	–		15	7.14	4.35
5	–		14	7.14	4.35
6	–		2		4.35
7	–		9	7.14	4.35
8	14	11.11	14	7.14	8.70
9	–		8	7.14	4.35
12	9	11.11	–		4.35
15	–		11	7.14	4.35
16	1,10,13	33.33	–		13.04
18	12,12	22.22	1,10,13	21.43	21.74
19	–		–		
22	–		4,5,6,7	28.57	17.40

“-” means no amplification or deletion

## Discussion

To elucidate the possible genetic reasons underlying the spontaneous abortion, we investigated chorionic villus specimens from early spontaneous abortion with no obvious abnormality using aCGH. Previously, the technique of G-binding karyotype analysis is regarded as the “Gold rule” of diagnosis of the chromosome abnormality, which can be used to test a set of chromosome number and obvious textural anomaly. However, this method has multiple limitations such as the failure of cell culture, time-consuming cell culture, microorganism pollution and the selective growth of maternal decidual cells. Besides, it has low resolution, hardly detects CNVs less than 5 Mb, and difficultly makes sure the size and breaking point of CNVs [20–24]. Other techniques, like fluorescence in situ hybridization (FISH) and CGH, are rarely applied due to their limitations. In contrast, CMA has the advantages including no need to culture cells, high throughput, high resolution and high accuracy, and it can scan the chromosomal non-equilibrium variations within the whole genome and detect CNVs at the submicroscopic structure level through one time hybrid experiment [25, 26]. Further, CMA improves diagnosis of chromosomal diseases at the genetic level avoiding the limitation of karyotype, FISH and CGH analysis techniques. aCGH analysis technology is mainly used in the diagnosis and research in cancers and genetic diseases [20, 21, 27, 28], but less in abortion. In this study, aCGH method is successfully used to determine submicroscopic chromosomal abnormalities in early spontaneous abortion patients.

Previous studies indicated that the submicroscopic chromosomal abnormalities is one of the major genetic causes of abortion and stillbirth [14, 25, 26]. Especially, stillbirth karyotypes are found at different trimesters. In 78 uncultured amniotic fluid specimens during second trimester, chromosomal abnormality is found using probes located at chromosome 13, 18, 21, X, Y. by FISH method [29]. Du et al. reported that absent fetal nasal bone and a higher rate of abnormal karyotype are included in the second trimester of pregnant women [30]. Microarray analysis is used to identiy stillbirths and provids a relative increase in the diagnosis of genetic abnormalities of compared to karyotype analysis [31]. Sahoo et al. [32] used a whole-genome SNP-based array (81.6%) and aCGH to determine the abnormalities in both fresh and formalin-fixed paraffin-embedded (FFPE) samples of products of conception (POCs) [33]. In this study, 15 submicroscopic chromosomal abnormalities are observed in early spontaneous abortion with using chorionic villus samples by aCGH method. Furthermore, micro-deletion/micro-duplication is observed in chromosomes 2, 4, 5, 6, 7, 8, 9, 12, 15, 16, 18, and 22, and uniparental disomy is found in chromosome 19. In addition, a total of 21 pathogenic CNVs including Waardenburg syndrome, Velocardiofacial/DiGeorge syndrome, Prader-Willi syndrome, Angelman syndrome, and Cornelia de Lange syndrome identified in this study are also reported in published articles [32, 34–37]. For instance, SPTAN1 gene deletion in chromosome 9:(130900001–133,080,000) has been reported to be associated with early infantile epileptic encephalopathy, infantile spasms, intellectual disability, and hypomyelination, and molecular defects of TOR1A gene lead to early-onset primary dystonia [31]. Besides, two novel CNVs on chromosomes 5 and 8 [chr5: (19080001–19,460,000) × 1, 380 K; chr8: (12540001–24,660,000) X3, 12.12 M] provide more possible causative CNVs in the development of abortion. Additionally, the spontaneous abortion alterations target certain chromosomes more than others. The CNVs of chromosomes 2, 16, 18, and 22 account for more than half of the cases (60.87%) in the present study.

## Conclusion

In sumarry, the aCGH analysis of 78 chorionic villus specimens shows that submicroscopic chromosomal imbalances might be one of the main reasons for early abortion. The chromosomal regions identified in this study may be critical in the development of abortion, and provides a basis for better understanding of the genetic cause of abortion. However, Further investigations are needed to including exploring the possible causative CNVs and genes including more samples and more comprehensive clinical information of the patients.

## Additional file

Additional file 1: **Figure S1.** The CNV plots of one patient are shown in **Figure S1** as a representative. (TIF 396 kb)

## Abbreviations

aCGH: Array-based comparative genomic hybridization
CGH: Comparative Genomic Hybridization
CMA: Chromosomal microarray analysis
CNVs: Copy number variations
FISH: Fluorescence in situ hybridization
SNP: Single nucleotide polymorphism
