# Supplementary figures and images for: Submicroscopic chromosomal imbalances contribute to early abortion
Source: Mol Cytogenet. 2018 Jul 21;11:41. doi: 10.1186/s13039-018-0386-0 (PMC6054741; doi:10.1186/s13039-018-0386-0)

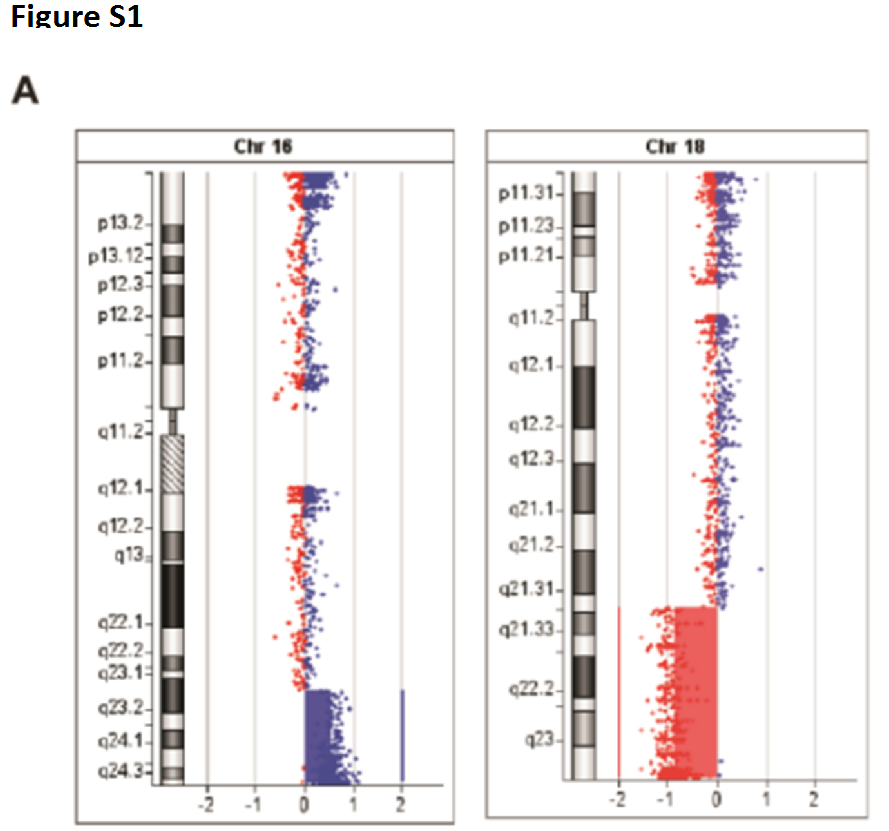

Supplement: Supplementary file 1 — Figure S1. The CNV plots of one patient are shown in Figure S1 as a representative. (TIF 396 kb) [file 13039_2018_386_MOESM1_ESM.tif]
